# The plasticity of cardiac sympathetic nerves and its clinical implication in cardiovascular disease

**DOI:** 10.3389/fnsyn.2022.960606

**Published:** 2022-09-09

**Authors:** Hideaki Kanazawa, Keiichi Fukuda

**Affiliations:** Department of Cardiology, Keio University School of Medicine, Tokyo, Japan

**Keywords:** heart failure, cardiac sympathetic nerves, parasympathetic nerves, plasticity, vagal nerve stimulation (VNS), cholinergic trans-differentiation, neuromodulation therapy

## Abstract

The heart is electrically and mechanically controlled by the autonomic nervous system, which consists of both the sympathetic and parasympathetic systems. It has been considered that the sympathetic and parasympathetic nerves regulate the cardiomyocytes’ performance independently; however, recent molecular biology approaches have provided a new concept to our understanding of the mechanisms controlling the diseased heart through the plasticity of the autonomic nervous system. Studies have found that cardiac sympathetic nerve fibers in hypertrophic ventricles strongly express an immature neuron marker and simultaneously cause deterioration of neuronal cellular function. This phenomenon was explained by the rejuvenation of cardiac sympathetic nerves. Moreover, heart failure and myocardial infarction have been shown to cause cholinergic trans-differentiation of cardiac sympathetic nerve fibers *via* gp130-signaling cytokines secreted from the failing myocardium, affecting cardiac performance and prognosis. This phenomenon is thought to be one of the adaptations that prevent the progression of heart disease. Recently, the concept of using device-based neuromodulation therapies to attenuate sympathetic activity and increase parasympathetic (vagal) activity to treat cardiovascular disease, including heart failure, was developed. Although several promising preclinical and pilot clinical studies using these strategies have been conducted, the results of clinical efficacy vary. In this review, we summarize the current literature on the plasticity of cardiac sympathetic nerves and propose potential new therapeutic targets for heart disease.

## Introduction

The pathophysiology of heart failure is complex, with a wide variety of causative diseases, including hypertension, valvular heart disease, ischemic heart disease, and cardiomyopathy. The heart has abundant sympathetic innervation, and basic and clinical studies have shown that cardiac sympathetic involvement is extremely important in the pathogenesis of heart failure (Cohn et al., 1984; Francis et al., 1990; Bristow et al., 1992). However, no clear conclusions have been reached regarding their detailed pathogenesis and significance. Recently, it has become clear that there is cross-talk between cardiomyocytes and cardiac sympathetic nerves mediated by various humoral factors. Additionally, molecular studies have revealed that sympathetic fibers are involved in the pathophysiology of heart failure, including axonal stretch, denervation, and functional changes, and that the cardiac sympathetic nerve itself has adaptive responses to anatomical and functional changes (Kimura et al., 2007, 2010; Kanazawa et al., 2010). In fact, excessive sympathetic activation and concomitant vagal withdrawal is a pivotal element in both the onset and the progression of cardiovascular diseases (Malliani et al., 1991; Malpas, 2010), and is linked to poor clinical outcomes and life-threatening complications (Grassi et al., 2015). Therefore, autonomic neuromodulation seems to be a key target in cardiovascular diseases, including heart failure, and device therapy to achieve autonomic modulation has garnered significant interest. Recently, promising neuromodulation therapies based on novel concepts that have the potential to lead to the development of new cardiovascular therapies and exploit sympathetic plasticity have emerged.

## Cardiac sympathetic nerve function and abnormalities in heart failure

In sympathetic nerve endings, norepinephrine (NE) is synthesized from tyrosine *via* dopamine through the action of tyrosine hydroxylase (TH) and dopamine β-hydroxylase (DBH), which are catecholamine synthases, and is stored in storage granules. Stimulation at higher levels induces exocytosis, which releases NE into the synaptic cleft. Binding of NE to β-adrenergic receptors on the myocardial cell surface produces positive potentiation and alteration effects. However, 95% of the released NE is taken up again by sympathetic nerve endings (uptake 1) and recycled. In addition, the released NE inhibits the release of NE by negative feedback *via* α2c receptors in sympathetic nerve endings (Small et al., 2002).

In heart failure, various abnormalities also occur in sympathetic nerve endings, with NE content in the myocardium decreasing until it is depleted as sympathetic activity is continuously increased. This has been explained by increased turnover of NE at sympathetic nerve endings and a decreased ability to retain NE due to excessive release (increased spillover) and impaired reabsorption (impaired uptake 1) (Chidsey et al., 1964). Furthermore, the expression of dopamine, the precursor of NE, and TH, the NE rate-limiting synthase, has also been found to be decreased, which has been attributed to anatomical sympathetic denervation itself (Himura et al., 1993) and clinically viewed as an ^123^I-meta-iodobenzylguanidine (MIBG) deficit.

Conversely, there are reports that although TH expression is decreased in animal models of heart failure, there are no quantitative changes in sympathetic nerve fibers and that functional changes are the primary focus of these changes (Liang et al., 2003).

As described above, various studies have demonstrated abnormalities of sympathetic nerve endings in heart failure. However, this pathophysiology is not fully understood as there are many inconsistencies, such as the fact that TH expression is decreased despite increased sympathetic nerve activity.

## Cross-talk between cardiac sympathetic nerves and humoral factors

In heart failure, the renin-angiotensin system is activated along with an increase in the release of endothelin (ET-1) and cytokines; this complex association of neurohumoral factor cross-talk is thought to modify the pathogenesis of heart failure along with cardiac sympathetic activation.

Nerve growth factor (NGF), also known as sympathetic nerve induction factor, belongs to the neurotrophin family and is important for neural differentiation, survival, and synapse formation (Snider, 1994). Importantly, the expression of NGF in target organs is thought to define sympathetic nerve fiber density (Heumann et al., 1984). It is well known that ET-1 expression is increased in cardiac hypertrophy and that NGF is specifically induced by this ET-1 in cardiomyocytes, indicating that the ET-1/NGF pathway is important for cardiac sympathetic innervation during heart development (Ieda et al., 2004).

Furthermore, in a rat model of right ventricular hypertrophy, it was reported that NGF protein expression was increased in conjunction with ET-1 mRNA expression in the right ventricle, and that sympathetic nerve fiber density in the right ventricle was also markedly increased (Kimura et al., 2007), indicating that the ET-1/NGF pathway is involved not only in cardiac development but also in pathological states. In contrast, in a model of severe end-stage heart failure with long-term exposure to high concentrations of NE, NGF expression in the myocardium was found to be reduced and sympathetic nerve fiber density decreased (Kimura et al., 2010).

Taken together, these results suggest that cardiac sympathetic nerve fiber density is strictly regulated by ET-1 expression and cardiac NGF expression under the influence of NE exposure, indicating that anatomical cardiac innervation is altered under various pathological conditions, known as “anatomical modulation”.

## Juvenile cardiac sympathetic nerves in the heart

Fetal-type genes such as beta-myosin heavy chain, skeletal muscle-type alpha-actin, ANP, and BNP are expressed in cardiomyocytes under the pathological condition of heart failure. This phenomenon is thought to be a defense mechanism by changing their isoforms. In recent years, it has become known that fetal-type genes are expressed not only in the heart but also in the nervous system as part of the body’s response to cellular damage.

Previous studies have assessed embryonic gene expression in cardiac sympathetic nerves using a model of right ventricular hypertrophy and right heart failure in which monocrotaline treatment of rats induced pulmonary hypertension (Kimura et al., 2007). Although myocardium-derived NGF expression was enhanced and cardiac sympathetic nerve fibers were overdistributed (hyperinnervation) in the hypertrophied heart, NE synthase TH and DBH activities were decreased, and sympathetic functions such as NE reabsorption also tended to decrease. Furthermore, these sympathetic nerve fibers were found to express (rejuvenate) markers of juvenile nerves, such as highly polysialylated neural cell adhesion molecule (PSA-NCAM). These results indicate that cardiac sympathetic nerve fibers in heart failure undergo juvenile degeneration, which may contribute to “functional modulation” of sympathetic function.

## Cytokine-induced neurotransmitter switching of sympathetic nerve

In 1974, it was reported that the addition of cultured myocardial supernatant to cultured sympathetic neurons induces differentiation of adrenergic sympathetic neurons into cholinergic neurons, i.e., switching the neurotransmitter from norepinephrine to acetylcholine in cultured neurons (Patterson and Chun, 1974). This phenomenon came to be called “neurotransmitter switching” or “cholinergic differentiation.” Furthermore, in 1989, the substance that induces this differentiation was identified as a leukemia inhibitory factor (LIF) (Yamamori et al., 1989). Subsequently, molecular elucidation of this receptor revealed that this factor belongs to the same family as a type of cytokine called IL-6. In addition to the IL-6 family members, ciliary neurotrophic factor (CNTF), oncostatin M (OSM), and cardiotrophin-1 (CT-1), the neurotrophin family members brain-derived neurotrophic factor (BDNF) and neurotrophin-3 (NT-3) are cholinergic differentiation factors that induce differentiation of adrenergic sympathetic nerves into cholinergic nerves, similar to LIF. It is said to be a retrograde signal from target organs that influences axon elongation, nerve survival, death, and synapse formation, as well as neurotransmitter function determination (Glebova and Ginty, 2005).

Thus, cholinergic differentiation is a phenomenon that has been confirmed *in vitro*, but the most studied representative example of cholinergic differentiation *in vivo* is the sweat gland (Francis and Landis, 1999; Stanke et al., 2006). The autonomic nerves innervating the sweat glands are anatomically sympathetic and express sympathetic markers such as TH during the first few days of life, while parasympathetic markers such as choline acetyltransferase (ChAT) are absent. However, when the sweat gland, the target organ innervated by this nerve, becomes functionally mature, an unknown cholinergic differentiation factor secreted by the sweat gland is transported retrogradely to the neuronal cell body *via* the gp130 receptor. Cholinergic differentiation then occurs in the neuronal cell body, which transiently exhibits both catecholaminergic and cholinergic properties. Furthermore, it eventually becomes cholinergic innervated, with acetylcholine acting as the neurotransmitter.

## Cholinergic trans-differentiation of cardiac sympathetic nerves

Although several cytokines and neurotrophic factors induce trans-differentiation of sympathetic neurons to cholinergic neurons *in vitro*, the physiological and pathophysiological roles remain unknown. Importantly, previous studies have found that cardiac sympathetic nerve fibers in heart failure patients and animal models can cause neurotransmitter switching and trans-differentiation from catecholaminergic into cholinergic neurons. This process is induced by gp130-mediated signaling *via* cholinergic differentiation factors released from a failing myocardium (Kanazawa et al., 2010). Moreover, increased inflammatory cytokines of the gp130 family, including IL-6, LIF, CT-1, and CNTF, in myocardial infarction also alter neurotransmitter switching in cardiac sympathetic nerves. This phenomenon suggests that sympathetic co-release of acetylcholine and NE may impair adaptation to a high heart rate and increase arrhythmia susceptibility (Olivas et al., 2016). These findings further indicate that the gp130 receptor-mediated action of cytokines secreted from the myocardium leads to bioprotective functional adaptations in a disease state.

## Heart failure and cardiac sympathetic nerves plasticity

**FIGURE 1:**
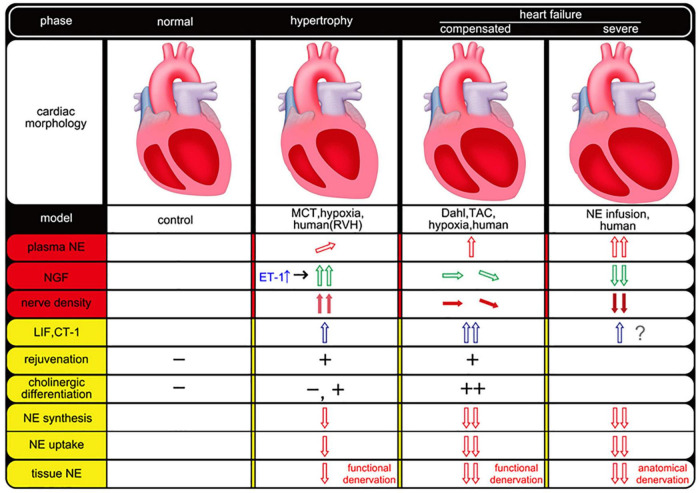
Temporal and spatial association of neurotrophic factors and cardiac sympathetic nerve plasticity in heart failure. The empty arrow indicates humoral factors and red means NE-related factors. Green (NGF) and blue (LIF, CT-1) arrows mean a different type of humoral factor (e.g., neurotrophic factor, gp130 cytokine family). The filled arrow indicates nerve density. This figure was created from the following references (Ieda et al., 2004; Kimura et al., 2007, 2010; Kanazawa et al., 2010). MCT, monocrotaline; RVH, right ventricular hypertrophy; TAC, transverse aortic constriction; NE, norepinephrine; NGF, nerve growth factor; ET-1, endothelin-1; LIF, leukemia inhibitory factor; CT-1, cardiotrophin-1.

Cardiac sympathetic and parasympathetic nerve fibers are embryologically identical in origin and are known to originate from neural crest cells. The functional changes in cardiac sympathetic nerve fibers observed in heart failure may capture their plasticity-based pluripotency (dedifferentiation and trans-differentiation). Furthermore, the viewpoint that this phenomenon acts in a bioprotective manner is a noteworthy concept that will greatly contribute to the elucidation of the pathogenesis of cardiac sympathetic nerve fiber abnormalities in heart failure. Anatomical changes in sympathetic density mediated by humoral factors after heart failure or myocardial infarction are also considered to be part of the plasticity of cardiac sympathetic innervation in pathological conditions (Oh et al., 2006). However, it is extremely difficult to explain this functional and anatomical change in cardiac sympathetic innervation in heart failure. Indeed, many factors likely underlie this complex pathophysiology, and it is assumed that a variety of phenomena can be observed over time and space, depending on the etiology, course of the condition, and differences in experimental animal models, further highlighting the complexity of the underlying mechanisms and phenomena (Figure 1).

## Neuromodulation therapy for cardiovascular disease

The state of increased sympathetic function observed during heart failure is one of the primitive and important compensatory mechanisms. However, this compensatory mechanism leads to further myocardial injury and impaired cardiac function. Additionally, it is the source of electrophysiologically lethal arrhythmias and a poor prognostic factor. Therefore, the body must have some compensatory mechanism to maintain myocardial protective and antiarrhythmic homeostasis, which might be an adaptive mechanism using plasticity.

**FIGURE 2:**
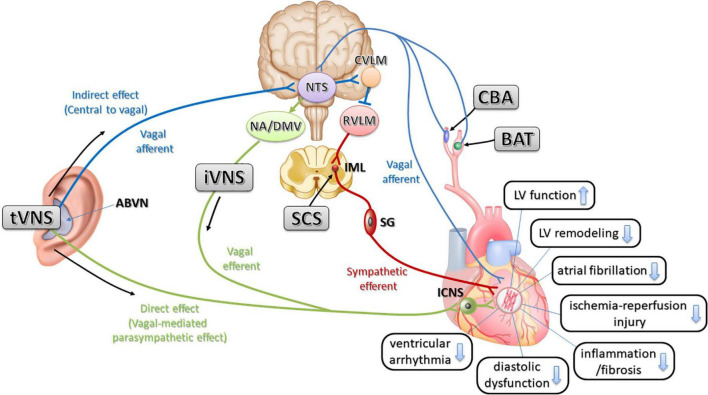
The relationship of physiological possible mechanisms of neuromodulation therapies and interactions for cardioprotective effects in cardiovascular disease. Stimulation of the ABVN increases input to the NTS in the medulla and influences the activity of NTS neurons projecting to the cardioinhibitory vagal efferent neurons of the DMV and NA (indirect effect). Stimulation of the ABVN may also excite NTS neurons, sending excitatory projections to the CVLM. The CVLM inhibits the RVLM, which is the primary source of excitatory drive to sympathetic preganglionic neurons. This inhibition then decreases sympathetic activity. NTS, nucleus tractus solitarius; ABVN, auricular branch of the vagus nerve; NA, nucleus ambiguous; DMV, dorsal motor nucleus of the vagus: RVLM, rostral ventrolateral medulla; CVLM, caudal ventrolateral medulla; tVNS, transcutaneous auricular vagal nerve stimulation; iVNS, invasive vagal nerve stimulation; LV, left ventricle; SG, stellate ganglion; ICNS, intrinsic cardiac nervous system; IML, intermediolateral nucleus; BAT, baroreceptor activation therapy; CBA, carotid body ablation; SCS, spinal cord stimulation.

Recently, interventional treatments in the autonomic nerves have been developed and are called neuromodulation therapies. These include baroreceptor activation therapy (BAT), spinal cord stimulation (SCS), and carotid body ablation (CBA), which use devices that block or denervate afferent nerve reflexes that increase sympathetic nerve activity, and vagal nerve stimulation (VNS) to increase parasympathetic activity, which could be direct or indirect. The promising data of these treatment methods are accumulated in preclinical and are expected to be clinically applied (Figure 2).

## Device-based sympathetic nerve activity suppression therapy

### Baroreceptor activation therapy

The arterial baroreceptor reflex is the most powerful regulatory system of the sympathetic nerve. By applying electrical stimulation to the baroreceptor near the carotid sinus, sympathetic nerve activity is reduced from the afferent pathway to NTS, and blood pressure lowering and deceleration effects can be expected. BAT was initially developed as a treatment for angina pectoris and hypertension, but it has been reported that NT-proBNP is lowered and exercise tolerance is improved for heart failure with reduced ejection fraction (Abraham et al., 2015; Schmidt et al., 2020). Currently, a clinical trial (The CVRx REBALANCE Registry) is underway to investigate the effect of the Barostim™ Neo system (CVRx, Minneapolis, MN, United States) on heart failure with left ventricular ejection fraction (LVEF) ≤35% (NCT04502316). It is necessary to evaluate the safety and effectiveness of the device in the future, but more recently, endovascular baroreflex amplification approaches have been developed as a less invasive approach. The MobiusHDTM device (Vascular Dynamics, Mountain View, CA, United States) is a nickel-titanium device that is implanted in the internal carotid sinus using a minimally invasive approach. A clinical trial (NCT04590001) is ongoing to evaluate the safety and effectiveness of the MobiusHDTM in patients with heart failure and reduced ejection fraction(≤40%).

### Spinal cord stimulation

Spinal cord stimulation (SCS) is a treatment method that stimulates the spinal cord by inserting a stimulation electrode into the epidural space and was originally developed for chronic pain syndromes and refractory angina pectoris (Brodison and Chauhan, 1999; Kemler et al., 2006). SCS suppresses sympathetic activity by modulating both afferent and efferent signaling between the spinal cord and heart, including the intrinsic cardiac nervous system (Ardell, 2016). In a preclinical study, SCS reduced infarct size, decreased ventricular arrhythmia, and improved LV systolic function in a canine model with myocardial infarction (Lopshire et al., 2009). However, DEFEAT-HF trials, which is a larger randomized study that enrolled heart failure patients with LVEF <35%, demonstrated no significant LV volume reduction (Zipes et al., 2016).

### Carotid body ablation

Chemoreceptor in the carotid body is central to the reflex mechanisms that primarily sense partial pressure of oxygen in arterial blood and control respiration and sympathetic nerve activity. It is known that this acceleration of the reflex mechanism induces sympathetic nerve activity in chronic heart failure. Reportedly, excision of the carotid body has the effects of lowering sympathetic nerve activity and blood pressure, improving cardiac function and prognosis in an experimental study (Fujii et al., 2017). As for a clinical application for heart failure patients, a first-in-man study to test the safety and feasibility of CBA in patients with heart failure demonstrated reduced sympathetic nerve activity and improved exercise tolerance (Niewinski et al., 2017). More recently, techniques for denervation by catheter ablation have been developed and are expected to be applied to patients with heart failure (Schlaich et al., 2018).

## Parasympathetic nervous system in cardiovascular disease

It has been reported that parasympathetic function is reduced in heart failure and myocardial infarction through changes in baroreflex sensitivity and heart rate variability (La Rovere et al., 2001; Florea and Cohn, 2014). Notably, the neurochemical phenotype and structural characteristics of intracardiac neurons, which include postganglionic parasympathetic neurons, are altered in cardiovascular disease. Additionally, the expression of choline acetyltransferase in intracardiac neurons is reduced significantly after myocardial infarction (Rajendran et al., 2016). The decreased parasympathetic function increases heart rate and acts as a compensatory mechanism for heart failure. However, it lowers the threshold for the development of lethal arrhythmias and is a poor prognostic factor. As a result, parasympathetic dysfunction contributes to the progression of heart failure and worsens the prognosis.

To date, M2 has been widely known as the muscarinic receptor distributed in the myocardium, but recently, it has been reported that M3 receptors are also present in the myocardium and their expression is increased during heart failure (Wang et al., 2004). Furthermore, it has been reported that stimulation of M3 receptors can act in a myocardial protective manner by signaling antioxidant, antiapoptotic, and protective mechanisms against ischemia (Yang et al., 2005). The mechanisms underlying this include acetylcholine inducing the expression of the hypoxia-inducible factor HIF-1α and reducing cellular damage by inhibiting apoptosis (Kakinuma et al., 2005) and possibly exerting its antiarrhythmic effects by improving connexin 43 function (Ando et al., 2005).

The above findings indicate that cardioprotective effects can be induced by directly or indirectly enhancing parasympathetic function in response to heart failure. These changes in muscarinic receptor expression during heart failure and the above-mentioned functional changes in cardiac sympathetic nerves to cholinergic nerves may also be considered to be purposeful changes.

## Therapeutic potential of vagal nerve stimulation for cardiovascular disease

It has been shown that activation of vagal function improves cardiac function, prevents remodeling, and improves prognosis after heart failure and myocardial infarction (Olshansky et al., 2008). In fact, preclinical studies have demonstrated the benefits of invasive VNS in reducing mortality, improving LV remodeling and hemodynamics, and reducing inflammation in various animal models of heart failure (Li et al., 2004; Zhang et al., 2009; Sabbah et al., 2011; Zhou et al., 2019).

Moreover, other preclinical VNS studies have demonstrated that VNS reduces ventricular arrhythmias and increases ventricular electrical stability in dog and pig models of myocardial infarction (Vaseghi et al., 2017; Nasi-Er et al., 2019). Interestingly, the potential underlying mechanisms identified included suppressing cardiac neuronal sprouting, inhibiting excessive sympathetic nerve sprouting, and pro-inflammatory responses by regulating gene expression (Zhao et al., 2021). Recently, findings from animal studies have suggested that the cardioprotective effects of VNS in myocardial infarction are provided by the activation of the cholinergic anti-inflammatory pathway on cardiac macrophages (Chung et al., 2020).

These emerging pieces of evidence of the cardioprotective effects of both invasive and non-invasive VNS suggest that this may be a feasible approach for treating cardiovascular diseases in humans.

## Clinical trials of vagal nerve stimulation

Several clinical trials following a first-in-human study (Schwartz et al., 2008) of a treatment method that electrically stimulates the vagus nerve in heart failure patients have already been conducted. Previous randomized clinical trials of VNS for heart failure with reduced ejection fraction (i.e., CARDIOFIT, ANTHEM-HF, NECTAR-HF, and INOVATE-HF) applied an invasive technique using an implantable pulse generator. These RCTs demonstrated that VNS leads to improvement of LVEF, NYHA (New York Heart Association) classification, and quality of life.

A recently published meta-analysis of RCTs with invasive VNS (iVNS) found that the intervention leads to a significant improvement in NYHA functional classification, quality of life, 6-min walking test distance, and NT-proBNP levels; however, they found that there was no difference in mortality (Sant’Anna et al., 2021). While iVNS has shown interesting results as an alternative therapeutic approach for heart failure, the invasiveness and cost of the surgical procedure, and poor patient tolerance limit the clinical applicability (Akdemir and Benditt, 2016).

Recently, transcutaneous stimulation of the auricular branch of the vagus nerve (tVNS) has attracted attention as an important non-invasive alternative approach with effects comparable to those of VNS with a surgically implanted device. Previous animal studies have shown that tVNS suppressed atrial fibrillation by down-regulating both c-fos and NGF and by preventing the loss of atrial connexins (Cx40 and Cx43), an essential component of gap junctions and a key player in the formation of AF substrate, which suggests that tVNS suppresses autonomic remodeling by modulating cardiac autonomic nervous system (Chen et al., 2015; Yu et al., 2017b).

Human studies of tVNS for cardiovascular disease are limited. A recent study assessing the effects of neuromodulation with tVNS observed a significant improvement in global longitudinal strain, inflammatory cytokines, and quality of life in patients with heart failure with preserved ejection fraction (Stavrakis et al., 2022). Alongside heart failure, in patients with paroxysmal atrial fibrillation (AF) who received tVNS, there was an 85% decrease in AF burden compared to the control group (Stavrakis et al., 2020). In addition, tVNS was found to reduce myocardial ischemia-reperfusion injury in patients with ST-segment elevation myocardial infarction (Yu et al., 2017a).

The hypothesis is that tVNS acts as a neuromodulation mechanism *via* not only direct efferent projections (vagal-mediated parasympathetic effect) but also indirect vagal afferent projections (central to vagal). The indirect effect of tVNS on the cardiovascular system acts through the nucleus tractus solitarius (NTS), the first synaptic station of the autonomic afferent projections in the central nervous system (Frangos et al., 2015). The recruitment of the NTS induced by tVNS likely also causes activation of excitatory inputs to the caudal ventrolateral medulla, which in turn inhibits the rostral ventrolateral medulla (RVLM), which is the primary source of excitatory drive to sympathetic preganglionic neurons. This inhibition would presumably lead to decreased sympathetic activity (Carandina et al., 2021) (Figure 2).

Taken together, tVNS has the potential to be a beneficial, simpler, and non-invasive therapy for cardiac autonomic tone modulation that can be used to treat cardiovascular diseases and a promising substitute for iVNS.

## Discussion

In this review, we have outlined recent concepts regarding the anatomical and functional changes of cardiac sympathetic nerves in heart failure and their relationship to the pathophysiology of heart failure. In addition, we highlighted the therapeutic potential of neuromodulation therapies, especially VNS, for cardiovascular disease. Charles Darwin once said that “It is not the strongest of the species that survives, nor the most intelligent that survives. It is the one that is most adaptable to change.”

We hope that further elucidation of the underlying molecular mechanisms combined with translational work investigating the cardioprotective effects of modulation of the autonomic nervous system, including tVNS in patients, will lead to new therapeutic strategies for cardiovascular disease.

## Author contributions

HK wrote the original manuscript. HK and KF reviewed and edited the manuscript. KF acquired funding. Both contributed to the article and approved the submitted version.
